# The impact of a blood-culture diagnostic stewardship intervention on utilization rates and antimicrobial stewardship

**DOI:** 10.1017/ash.2023.304

**Published:** 2023-09-29

**Authors:** Kelvin Zhou, Melinda Wang, Sabra Shay, James Herlihy, Muhammad Asim Siddique, Sergio Trevino Castillo, Todd Lasco, Miriam Barrett, Mayar Al Mohajer

## Abstract

**Background:** Blood cultures are often ordered when an infection is suspected; however, they have a low yield in most cases. The overuse of blood culture is associated with high contamination rates, resulting in excess diagnostics, unnecessary antibiotics, longer hospital stays, and higher hospital costs. We evaluated the safety of a multifaceted intervention, which encompassed education and blood-culture restriction, and its impact on blood-culture utilization and antibiotic use in adult intensive care unit (ICU) patients. **Methods:** The study was performed between October 2020 and October 2021 in the 12 general medicine and specialty ICUs of a quaternary academic care center. The intervention, implemented in April 2021, included providing education to ICU and infectious disease physicians based on an algorithm adapted from the Johns Hopkins DISTRIBUTE study in addition to restricting blood-culture ordering on these units to these providers. The month of April 2021 was excluded as a washout period. Study outcomes comprised blood-culture utilization, blood-culture positivity, days of therapy (DOT), and length of therapy (LOT), which were compared across the study periods using IRR or the Pearson χ^2^ test, as appropriate. In addition, 30-day mortality and 30-day ICU readmission were evaluated utilizing multiple COX regression models. **Results:** In total, 6,303 patients (2,087 MICU, 3,636 SICU, and 580 both) were included in the study, with a median age of 65 years (IQR, 21). Most participants were male (57.5%), with a median length of stay of 175 hours (IQR, 186). After the intervention, blood-culture utilization rates decreased from 15.4% to 12.4% (IRR 0.80, 95% CI, 0.76–0.85) (Fig. 1). There was no difference in blood-culture positivity between the preintervention period (11.05%) and the postintervention period (11.64%; *P* = .459). Days of therapy decreased from 1,180 to 1,130 per 1,000 patient days (IRR, 0.96; 95% CI, 0.95–0.98), and the length of therapy decreased from 602 to 579 per 1,000 patient days (IRR, 0.96; 95% CI, 0.94–0.99) (Fig. 2). There was no difference in 30-day mortality (*P* = .241) nor 30-day ICU readmission (*P* = .888) across the study periods after adjusting for potential confounders (Table 1). **Conclusions:** Our multifaceted intervention decreased blood-culture and antimicrobial utilization in the ICUs without significantly affecting the positivity rate, mortality, or readmission. This study suggests that educating providers on appropriate blood-culture use along with restriction could safely improve healthcare outcomes. Further studies are warranted to validate our results across various institutions and to evaluate the impact of blood-culture optimization in non-ICU patients.

**Figure:**
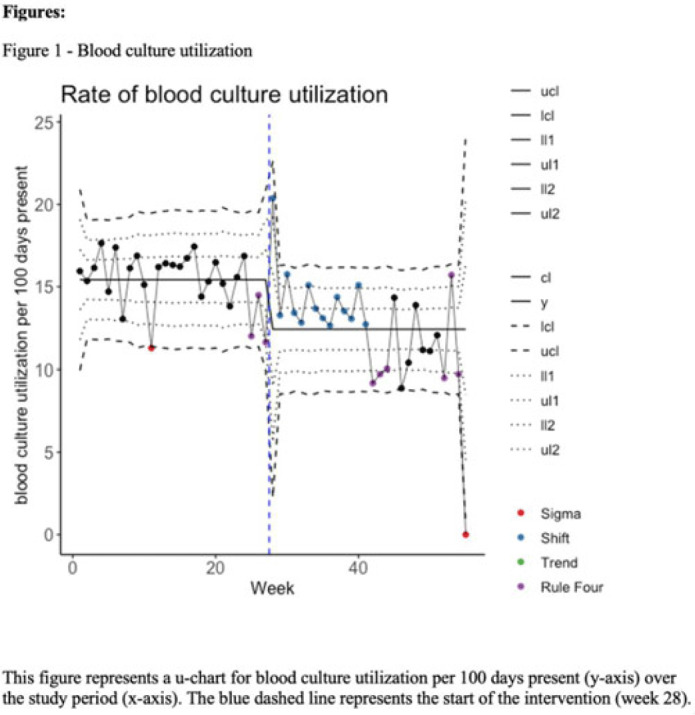

**Figure:**
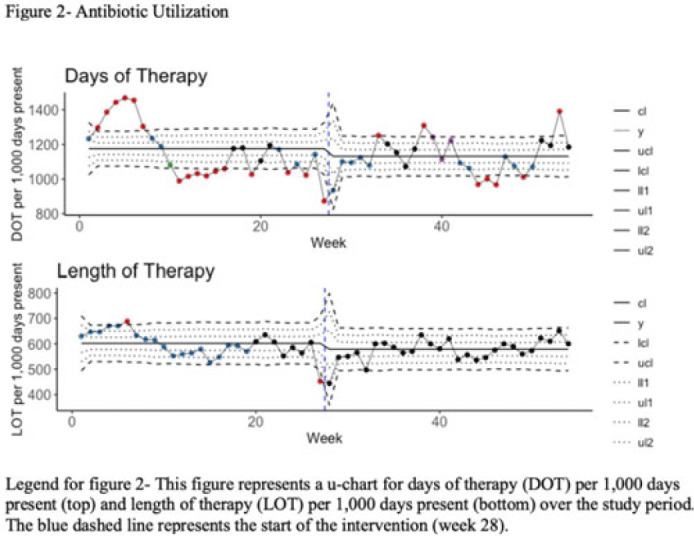

**Figure:**
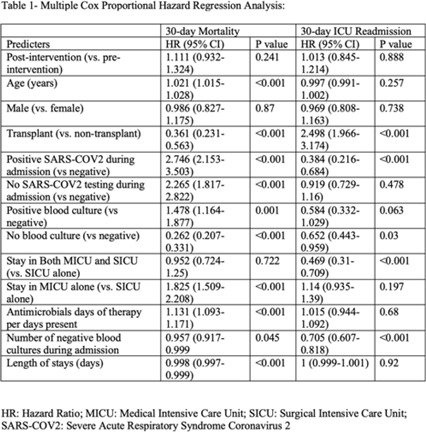

**Disclosures:** None

